# Neurophyisological and Neurocognitive Endophenotypes for Schizophrenia Genetics Research

**DOI:** 10.4306/pi.2008.5.4.199

**Published:** 2008-12-31

**Authors:** Yeon Ho Joo

**Affiliations:** Department of Psychiatry, University of Ulsan College of Medicine, Asan Medical Center, Seoul, Korea.

**Keywords:** Genetics, Schizophrenia, Endophenotype

## Abstract

There is growing interest in the genetic analysis of schizophrenia using endophenotypes rather than clinical diagnosis or symptom dimensions. Endophenotypes could be alternative phenotypes for the clinical phenotypes. With their intermedicate and quantitative characteristics, endophenotypes could be functionally important links in the pathways between the genetic variation and clinical expression of the disorder. In this regard, the neurophysiological and neurocognitive endophenotypes used in the genetic analysis of schizophrenia have been reviewed.

## Concept for Endophenotype

Genetics is all about the relationship between the underlying genotype and manifested phenotypes. The hunt for disease-related genes has proven to be more difficult than was initially anticipated and has been successful mostly in disorders that are clearly defined and homogenous. Psychotic disorders have posed a particularly difficult problem, due to the lack of preciseness in defining the boundaries of the clinical phenotype and the absence of objective, biological tests that confirm the diagnostic categorization. In complex diseases such as schizophrenia, there might be a long and winding pathway between the candidate genes and the clinical diagnosis. In schizophrenia, a large amount of genetic studies have been done to elucidate the susceptibility genes that underlie the phenotype. Until now, there have been no widely accepted susceptibility genes that are related to schizophrenia, even though there is well established evidence that schizophrenia has substantial genetic components.

This is where the concept of the endophenotype comes in. This concept was first introduced into the schizophrenia literature by Gottesman and Shields in 1972 and was designed to signify quantitative measures that were "intermediate", functionally important links in the pathways between genetic variation and the clinical expression of the disorder.^1^ Other terms have been used interchangeably, such as the intermediate phenotype, biological marker, subclinical trait, and vulnerability marker. These terms may not necessarily reflect genetic underpinnings, but rather associated findings.

Other fields of medicine have had some success in using endophenotypes for genetic studies. For example, the multiple genes causing long QT syndrome were identified by using QT elongation, as measured by electrocardiogram (ECG), rather than by using the phenotypes of syncope, ventricle arrhythmias, and sudden death.^2^,^3^

One strategy to dissect the complex nature of schizophrenia is the analysis of the quantitative neurobiological deficits, endophenotypes, that are present in schizophrenia patients and their first-degree biological relatives.^4^ As relatively simple, quantifiable biobehavioral characteristics, individual endophenotypes are presumably determined by fewer genes than the more complex phenotype of schizophrenia. Endophenotypes were originally defined as measurable components unseen by the unaided eye along the pathway between the disease and distal genotype.^4^

An endophenotype-based approach has the potential advantage of facilitating the genetic dissection of schizophrenia, because they would ideally have monogenic roots. In reality, however, an endophenotype is a rather ill-defined construct that probably involves neurophysiological, biochemical, endocrinological, neuroanatomical, cognitive and even neuropsychological factors.^5^ Hence, the use of endophenotypes needs to be tempered by the appreciation that without controls and limits, their usefulness may be obscured. A putative endophenotype does not necessarily reflect genetic effects. Indeed, these biological markers may be environmental, epigenetic, or multifactorial in origin. However, it psychiatric diagnoses could be decomposed or deconstructed, I could render the genetic analysis more straightforward.

## Criteria for Endophenotypes

A number of criteria for evaluating the validity of candidate endophenotypes have been suggested.^4^,^6^-^9^ Among them, the following criteria have been the most frequently adopted.

1. The endophenotype should be heritable.
2. The endophenotype should be state-independent.
3. The endophenotype should be associated with illness in the population.
4. Within families, endophenotype and illness should co-segregate.

The final criterion for an endophenotypic marker has two distinct components. First, the marker must co-segregate with the illness within a family. The second component specifically relates to diseases with complex inheritance patterns. In this case, unaffected family members should show some level of disruption on the marker compared with the population at large.^10^

In psychiatric fields, a number of attempts have been made to develop and determine the feasibility of candidate endophenotypes. However, few have met all the criteria listed until now.^4^

## Neurophysiological Endophenotypes

The ideal neuropsysiological endophenotype is one that exhibits a robust and stable deficit in both patients and unaffected family members and shows strong evidence of both heritability and co-segregation with illness within pedigrees. It should also be easily and rapidly measured with minimal demands on the subject, demonstrate excellent test-retest and across-site reliability and, preferably, reflect a discrete neurobiological mechanism that is both informative for the pathophysiology of the disorder and regulated by a limited number of genes.^11^

Inhibitory deficits in schizophrenia have been recognized since long ago.^12^ Patients with schizophrenia are unable to screen out trivial stimuli and focus on salient aspects of the environment. Patients with schizophrenia exhibit deficits in several neurophysiological measures of information processing that have been proposed as candidate endophenotypes. Measures of inhibitory failure include prepulse inhibition of the startle reflex, P50 auditory evoked potential suppression, and antisaccade eye movements. For example, the neurophysiological measures in Consortium on the Genetics of Schizophrenia (COGS) include P50 auditory evoked potential suppression, prepulse inhibition (PPI) of the startle reflex, and antisaccade (AS) eye movements. Measures of impaired deviance detection include mismatch negativity and the P300 event-related potential.

The p50 suppression test uses two auditory stimuli presented at 500-msec intervals. The P50 wave is a midlatency auditory evoked potential that exhibits reduced amplitude, or suppression, when a second click sound is presented 500 ms after the initial click. Freedman et al. identified p50 suppression as an important endophenotype of schizophrenia.^13^ The use of p50 suppression as a candidate endophenotype for genetic studies is further supported by the identification of significant linkage of p50 suppression with a genetic marker in the promoter region of the alpha-7 subunit of the nicotinic receptor.^14^

PPI is the normal reduction in startle that occurs when a starting stimulus is preceded 30-300 ms by a weak prestimulus.^15^ Prepulse inhibition is a generally conserved finding among vertebrates and, as such, it has been the target of several rodent studies.^4^ It is also deficient in patients with schizophrenia and their relatives.^16^,^17^ Abnormal prepulse inhibitions were found in obsessive compulsive disorder^18^ and Huntington's disease,^19^ among others. Elements of the cortico-striato-pallido-thalamic circuitry in humans and animal models are known to be implicated in the neural regulation of PPI.^20^

The saccadic performance in schizophrenia patients is characterized by an increased proportion of antisaccade errors.^21^ In their more proximal connection to neuronal mechanisms, neurophysiological endophenotypes are a much stronger signal for the putative disease-related genes, compared to more variable and complex clinical phenotypes.^22^

Neurophysiological measures used in the genetic study of schizophrenia are summarized in Table 1.

## Neurocognitive Endophenotype

Cognitive impairments have been considered core features of schizophrenia since the time of Bleuler. Cognitive measures along with neurophysiological measures are highlighted as endophenotypes for schizophrenia recently. There is substantial evidence that measures of sustained attention or vigilance, verbal declarative memory and working memory are valid endophenotypes in schizophrenia.

The Continuous Performance Test (CPT), as neurocognitive measure, is used in the COGS for the measurements of both verbal memory and working memory.^23^ Actually, CPTs are most widely used measures of deficits in sustained attention. Among them, the Degraded Stimulus Continuous Performance Test (DS-CPT) and Continuous Performance Test, Identical Pairs version (CPT-IP) are most sensitive tools. These 2 measures have shown potential stability over time. CPT impairments are present in schizophrenia even in a clinically remitted state. CPTs with high perceptual discrimination loads or working memory loads also have been used successfully for the detection of neurocognitive deficits among biological relatives of patients.

Deficits in verbal declarative memory (VDM) are one of the most prominent cognitive impairments in schizophrenia. The Wechsler Memory Scale, 3^rd^ edition (WMS-III), test of story recall (Logical memory, LM)^24^ and the California Verbal Learning Test, 2^nd^ edition (CVLT-II), are the most widely used neurocognitive tests.^25^

Working memory (WM) deficits have been described as core cognitive features of schizophrenia.^26^ They have also been related to the clinically important features of schizophrenia, such as poor social and vocational function.^27^,^28^ Diverse paradigms and specific tests have been used to access the construct of working memory. Although the heritability estimates of working memory tests do not show robust results,^29^ working memory deficits seem to reflect state independent features that are not due to potential compounds. The Letter-Number Sequencing task (LNS)^24^ has been selected by the COGS. The LNS and related verbal span tasks have been successfully implemented in large, multicenter studies of schizophrenia patients.^30^

Neurocogntive construct and measures most frequently used in the genetic study of schizophrenia are summarized in Table 2.

## Conclusion

The endophenotype strategy could be a powerful and effective means of identifying vulnerability genes in schizophrenia. There is, however, little convincing evidence yet that the genetic architecture of the endophenotypes is substantially simpler than that of the schizophrenia itself. Many of the endophenotypes remain complex, thereby decreasing their utility in a genetic study. Further studies need to be done before endophenotypic measures can be used in the genetic analysis of schizophrenia.

## Figures and Tables

**TABLE 1:**
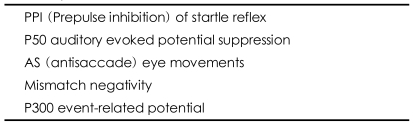
Neurophysiological measures used in the genetic study of schizophrenia

**TABLE 2:**
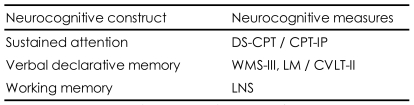
Neurocogntive construct and measures used in the genetic study of schizophrenia

DS-CPT: Degraded Stimulus Continuous Performance Test, CPT-IP: Continuous Performance Test, Identical Pairs version, WMS-III, LM: The Wechsler Memory Scale, 3^rd^ edition, test of story recall, Logical memory, CVLT-II: California Verbal Learning Test, 2^nd^ edition, LNS: Letter-Number Sequencing task
